# Humeral Diaphyseal Fracture Nonunion: An Audit of the Outcome from Intramedullary Nailing and DCP Plating

**DOI:** 10.1155/2019/9107898

**Published:** 2019-07-22

**Authors:** Ivan Micic, Erica Kholinne, Jae-Man Kwak, Yucheng Sun, Akriti Nanda, Hyojune Kim, Kyoung-Hwan Koh, In-Ho Jeon

**Affiliations:** ^1^Clinic for Orthopaedic Surgery and Traumatology, Clinical Center Nis, Nis, Serbia; ^2^Department of Orthopedic Surgery, St. Carolus Hospital, Jakarta, Indonesia; ^3^Department of Orthopedic Surgery, Asan Medical Center, University of Ulsan College of Medicine, Seoul, Republic of Korea; ^4^Department of Hand Surgery, Affiliated Hospital of Nantong University, Nantong, Nantong University, Jiangsu, China

## Abstract

**Purpose:**

This study aims to compare the functional outcomes of nonunion humeral diaphyseal fractures following conservative treatment when managed surgically with either a plate or intramedullary (IM) nail fixation.

**Methods:**

This was a retrospective study of 56 patients with nonunion humeral fractures following conservative treatment who underwent plate or IM nail fixation between 2007 and 2014. Comparison was made for short term profile (intraoperative blood loss, duration of surgery, and length of hospitalization) and long term clinical outcome with functional score (Constant-Murley score (CMS)) and Disabilities of the Arm, Shoulder and Hand (DASH) score). The union and complication rate were also compared.

**Results:**

There were 36 and 20 patients included in plate and IM nail fixation group with the average of 36.14 ± 7.54-month follow-up time. The intraoperative blood loss, duration of surgery, and length of hospitalization were superior in IM nail group compared to plate group (p < 0.001, p < 0.001, and p < 0.001, respectively). The mean CMS and DASH score were superior in the plate group compared to the IM nail group (82.40 ± 16.84 versus 77.58 ± 12.96; 17.46 ± 11.05 versus 20.86 ± 11.63, respectively; with p = 0.246, p = 0.299, respectively). Plate fixation group showed higher union rate and complication rate compared to IM nail group (100% versus 90%, 13.8% versus 10%, respectively).

**Conclusions:**

IM nail and plate fixation demonstrated comparable clinical outcome. IM nail fixation showed superior short term result with lower complication rate which benefits the elderly group patients with significant comorbidities.

## 1. Introduction

A nonunion of the humeral shaft is not unusual in clinical practice and is a complication of both conservative and operative treatment [1, 2]. Multiple factors contribute to nonunion rates. Higher incidence of nonunion is related with open fractures, high-energy injuries, bone loss, soft tissue interposition, unstable fracture patterns, insult to blood supply, anf infection [3].

Nonunion of the humeral shaft will result in debilitating pain with significant functional loss resulting in long absence of work and life quality impairment. Such condition require operative treatment to achieve adequate fixation often complicated by infection, prior surgical history, and significant bone loss [4–6].

Various surgical treatments have been reported for nonunion of the humeral shaft which include plating and intramedullary nail (IM nail) fixation with or without biologic augmentation [1, 7]. Previous study has reported 91 % of union rate when plate fixation was used to treat nonunion of the humeral shaft [1]. Plate fixation has several benefits such as providing absolute stability with perfect reduction, the ability to take down interpositional nonunion tissue and to explore radial nerve if necessary, facilitate the application of bone graft, and prevent any injury to shoulder soft tissue. IM nail fixation was reported to achieve 100% union rate in the previous study [8]. IM nail has been known to have several benefits from its relative stability characteristic such as minimal soft tissue dissection and ability to provide adequate reduction. However, it also has several drawbacks such as less perfect reduction with higher risk of distraction, inability to take down the interpositional nonunion tissue, higher risk of having radial nerve injury due to its inability to visualize the nerve, and technical difficulty to pass the guide rod and may insult the soft tissue around shoulder joint.

While the advantages and disadvantages of plate and IM nailing fixation have been reported, there has been no report comparing the clinical results between the both fixations for the treatment of humeral shaft nonunion. The aim of current study was to compare the functional outcome between plate and IM nailing fixation to treat nonunion of humeral shaft fractures following conservative treatment.

## 2. Methods

### 2.1. Patient Selection

Data from patients who underwent surgical treatment for a nonunion humeral shaft fracture at a tertiary referral hospital in Serbia between January 2007 and December 2014 were retrospectively evaluated. Humeral shaft nonunion was defined as a fracture that showed minimal healing 6 months after initial conservative treatment. The initial conservative treatments were hanging cast, brace, and sling. A diagnosis of nonunion was based on clinical examination (local tenderness), false motion, and plain radiograph imaging of the affected extremity. Nonunion type was defined according to the criteria of Weber and Cech [9].

Exclusion criteria were made for nonunion fractures following an open humeral fracture or after surgical treatment, nonunion fractures associated with infection and immunocompromised condition, coexisting radial nerve palsy. From January 2007 to January 2012, 36 patients were treated with plate fixation; from February 2012 to December 2014, 20 patients were treated with IM nailing, by a single senior orthopaedic trauma surgeon. Plate fixation was performed using a narrow 4.5-millimeter Dynamic Compression Plate (Synthes, Paoli, PA, USA), and IM nail fixation was performed using a locking intramedullary nail system (Stryker, Michigan, IN, USA). This change in practice was made by the treating surgeon because of the perceived potential cost benefits and reduced operating time using the IM nail, although these had not been quantified formally before the change was made.

Medical records were reviewed for demographics data (age, sex, and dominant arm), type of nonunion, time from injury to surgery, short term profile (intraoperative blood loss, duration of surgery, and length of hospitalization), follow-up time, and complication rate. The pre- and postoperative radiographic images were reviewed by independent surgeons. Postoperatively, all patients followed a standardized rehabilitation program with arm immobilization for 3 weeks. Active exercise was permitted 3 weeks after surgery, followed by a gradual increase in exercise level. Each patient was followed up in the outpatient clinic every 4–6 weeks, where they underwent clinical examination, plain radiograph imaging, and calculation of Constant-Murley score (CMS) and Disabilities of the Arm, Shoulder and Hand (DASH) score.

Follow-up was continued until fracture union was achieved, which was defined radiologically as the presence of cortical bridging at 75% of the circumference of the bone and clinically as the absence of discomfort at the nonunion site. All patients were followed up for at least 24 months. The union rate and time for each group were recorded.

### 2.2. Statistical Analysis

Test for normality using Kolmogorov-Smirnov was applied to all data set prior to statistical analysis. Mann-Whitney U test was used to compare data set with skewed distribution; meanwhile independent t-test was used to compare data set with normal distribution. Skewed distribution data value was expressed with median and range while normal distributed data value was expressed with mean and standard deviation. Significance level was set at p < 0.05. Statistical analysis was performed with Statistical Package for the Social Sciences software (v. 12.0; SPSS, Inc., Chicago, IL, USA). Statistical analysis was conducted with the supervision by professional biostatistician.

## 3. Results

### 3.1. Patients Demographics

Table 1 showed the patient demographics of both groups. A total of 56 patients (26 male, 30 female) with a mean age of 58.9 years (range, 21–83 years) were included in the analysis. Eleven patients had oligotrophic nonunion and 45 had hypertrophic nonunion type. Thirty-six patients underwent plate fixation (plate group) while 20 patients underwent IM nail fixation (IM nail group). The plate group comprised 17 male and 19 female patients with an average age of 53.25 years (range, 21–79 years). The IM nail group comprised 9 male and 11 female patients with an average age of 62.16 years (range, 21–83 years). The dominant arm was involved in 24 patients, 15 from the plate group and 9 in the IM nail group. There was no significant difference regarding patient demographic between both groups.

### 3.2. Short Term Profile

Table 2 showed the comparison of intraoperative characteristics. The time from injury to surgery for all patients was 31.43 months (range, 24–48 months). No significant differences were seen between the two groups in terms of interval from injury to surgery. The average intraoperative blood loss was significantly greater in the plate fixation group than the IM nail group (p < 0.001). The average duration of surgery was significantly shorter in the IM nail group than the plate fixation group ( p < 0.001). The average length of hospitalization period was also significantly shorter in the IM nail group than the plate fixation group (p < 0.001).

### 3.3. Postoperative Outcomes

Postoperative outcomes are summarized in Table 3. There was no significant difference for follow-up time in both groups (p = 0.06). The union rate for plate group was 100%. The union rate for IM nail group was 90% with 2 female patients developed nonunion at the period of follow-up. The union time for plate group was faster (4.24 ± 0.67 months) compared with IM nail group (4.47 ± 0.60 months) with no significant statistical difference (p = 0.976). One female patient from IM nail (age 75 years) which failed to achieve union following surgical management did not received further surgical treatment as her general condition did not allow any additional surgery under general anesthesia. The other female patient from IM nail group (age 76 years) which failed to achieve union was treated with a narrow, 4.5-millimeter titanium limited—contact dynamic compression plate (Synthes, Paoli, PA, USA) with the augmentation of autologous cancellous bone graft from iliac crest as described by Ring et al. [1]. During the additional surgery, the fracture site was found to have atrophic nonunion type with the development of pseudo-arthrosis. The additional surgery included the removal of hardware, debridement of avascular bone which resulted in 2cm humeral shortening. There was no evidence of local and systemic infection for this patient.

Clinical function was assessed using the CMS and DASH scores. According to the CMS, 38.9% of patients (n = 14) in the plate group had excellent or good outcomes compared with 20% (n = 4) in the IM group. Satisfactory results were seen in 22.2% (n = 8) of patients in the plate group and 55% (n = 11) in the IM nail group. The mean CMS was superior in the plate fixation group (82.4 ± 16.84) than in the IM nail group (77.58 ± 12.96) with no significant statistical difference (p = 0.246). The mean DASH score was superior in the IM nail group (20.86 ± 11.63) than in the plate fixation group (17.46 ± 11.05) with no significant statistical difference (p = 0.299).

### 3.4. Complications

The complication rate for the series was 16.07% (9 of total 56 patients). Transient radial nerve palsy occurred in five patients treated with plate fixation; all cases resolved spontaneously within less than 6 months. Fracture of the distal humeral segment during IM nail placement was noted in two patients. The complication rate for plate fixation group (13.8%) was higher than IM nail group (10%) with no significant statistical difference (Table 3).

## 4. Discussion

Reports of humeral shaft nonunion in the literature focus largely on aseptic nonunion. Although several surgical methods are used in the treatment of humeral shaft nonunion, including open plating, IM nailing, and external fixation, very few studies have directly compared the outcomes associated with these different approaches especially for nonunion following conservative treatment.

The result of current study shows 2 major important findings as follows: (1) surgical treatment for nonunion humeral diaphyseal fractures following conservative treatment resulted in favorable outcome and (2) the functional result between plate fixation and IM nail fixation for treating nonunion humeral diaphyseal fractures following conservative treatment is comparable. The additional findings of the current study are (1) the superiority of short term profile for IM nail fixation group compared to plate fixation group and (2) the average complication rate for IM nail group lower than those observed in plate fixation group. The short term profile includes the intraoperative blood loss, duration of surgery, and length of hospitalization.

The union rate and time of plate fixation group were superior compared to IM nail group. We postulated that the higher number hypertrophic nonunion type in the plate fixation group contributed to this result. A recent systematic review of union rates after the surgical treatment of humeral shaft nonunions showed high healing rates in patients who underwent plate fixation with autologous bone grafting (98%) or plate fixation without bone grafting (95%). By contrast, union rates were lower in patients undergoing revision surgery IM nailing (88% for IM nailing with autologous bone graft and 66% for IM nailing without autologous bone graft). An alternative approach, external fixation, also yielded a high healing rate of 98% but was associated with the highest rate of complications [10].

In the current study, the interval between injury and surgery was variable but the mean value in each group showed no significant difference. When comparing intraoperative factors, IM nail fixation was associated with a significantly shorter surgery time, approximately half the duration required for plate fixation. Patients treated with IM nail also had smaller incisions, minimal soft tissue trauma, and minimal blood loss. Therefore, the duration of hospital stay was seen to be significantly lower in the IM nail group. The advantages of this approach could be of particular importance in patients for whom more intense surgery and a prolonged hospital stay would be detrimental. This may be particularly important in elderly patients or those with significant comorbidities.

When comparing postoperative outcomes between the treatment groups, no statistically significant difference was seen in union time, demonstrating that both approaches were associated with a high rate of success. Sufficient stability was achieved even in osteoporotic bones using either a plate or IM nail. In terms of the postoperative functionality scores, the plate fixation group demonstrated a superior CMS and DASH score than the IM nail group, although no significant differences were seen in statistical test.

No significant difference in the number of complications was seen between the two groups although plate fixation showed higher complication rate (13.8%) compared to IM nail group (10%). In the plate fixation group, the most common complication was transient radial nerve palsy with all cases resolved spontaneously within 6 months. In the IM group, the most notable complication was fracture of the distal humeral segment during nail placement. This finding is correlated with the previous report that described the iatrogenic radial nerve injury following surgical intervention is between 2% and 5% [11]. We believed that this occurred due to 2 factors which are the vulnerability of radial nerve itself and the manipulation during nonunion surgery. Abundant scar tissue was usually encountered in the nonunion surgery with open manner (plate fixation). This will result in the higher risk of having adjacent neurovascular injury. Our study also showed 2 patients with distal humeral fracture in the IM nail group. These 2 patients belong to the geriatric population (age 75 and 80 years). Previous study had reported the intraoperative complications for distal humeral fracture most frequently occurred in osteoporotic bone. The incidence reported in the previous study is as high as 2.7% [12]. We think that the geriatric background of our two patients contributed to this complication. Although we admitted that no bone density investigation was performed prior to the surgery. The distal humeral fracture seen in the IM nail group was found to be stable which does not require a change in the treatment method or supplementary fixation following the previous study recommendation [12].

The study has a number of limitations that should be considered. First, the small sample size and single-center design means that firm conclusions cannot be drawn and further investigation is required in a large, randomized study or a multicenter trial. Secondly, the study inclusion criteria were relatively stringent, evaluating only patients with midshaft nonunion and excluding those complicated with infection or a significant bone defect. As the clinical course of septic nonunion differs in many respects from that of simple nonunion, we considered that it would not be possible to compare all the potential variables in this study. Similarly, patients with a large bone defect were excluded as this often requires special techniques to achieve union, such as vascularized bone grafts, distraction histogenesis, or different plates [2, 9, 13, 14]. Thirdly, our retrospective design and imbalance sample number between both groups also contributed to the study limitation. Fourthly, the plate fixation group was younger than the IM nail group which susceptible to retrospective selection bias. However, the impact of bias will be minimized given the tendency to favor IM nail group in the result.

It is evident that surgical approaches using plate and IM nail techniques are both associated with a range of advantages and disadvantages. The plate technique is associated with a good degree of stability, while the IM nailing technique has intraoperative advantages, such as limited exposure to the nonunion site with minimal surgical trauma to the soft tissue, minimal blood loss, and a short surgical duration, resulting in shorter postoperative hospital stays. This may be particularly important in elderly patients or those with significant comorbidities. In our series, IM nailing and plating both had satisfactory and comparable healing rates with good functional results and minimal complications. Therefore, we can conclude that both approaches can be considered for fixation in humeral shaft nonunion. The decision regarding which technique to use is likely to be based on the intraoperative details, in consideration with the patient's status and the surgeon's preference and experience.

## 5. Conclusions

Our retrospective series showed that plate and IM nail fixation had satisfactory and comparable healing rates with good functional results and minimal complications. We recommend the use of IM nail fixation for elderly patients or those with significant comorbidities for its shorter surgical duration, lower intraoperative blood loss, and complication rate.

## Figures and Tables

**Table 1:** Patient demographics.

Variable	Plate Fixation Group N = 36	IM Nail Fixation Group N = 20	p-value
Sex (%)	Female: 19 (52.8%) Male: 17 (47.2%)	Female: 11 (55.0%) Male: 9 (45.0%)	0.876
Age, years (Median, Range)	53.25 (21 – 79)	62.16 (21 – 83)	0.06
Dominant arm involvement, n (%)	15 (41.6%)	9 (45%)	0.813
Type of nonunion, n (%)	Oligotrophic: 10 (27.7%) Hypertrophic: 26 (72.3%)	Oligotrophic: 5 (25%) Hypertrophic: 15 (75%)	0.252

Statistical analysis was done with Mann Whitney u test with p < 0.05 being considered significant.

**Table 2:** Comparison of intraoperative characteristics between both groups.

Variable	Plate Fixation Group N = 36	IM Nail Group N = 20	p-value
Time from injury to surgery, month. (median, range)	31.00 (24 – 48)	31.66 (24 – 40)	0.643
Intraoperative blood loss, ml. (median, range)	97.50 (50 – 150)	62.92 (25 – 100)	< 0.001*∗*
Duration of surgery, minutes (mean ± SD)	73.25 ± 16.32	39.02 ± 11.00	< 0.001*∗*
Length of hospitalization, days (median, range)	3.90 (2 – 6)	1.16 (1 – 2)	< 0.001*∗*

SD = Standard Deviation.

Time from injury to surgery, intraoperative blood loss, and length of hospitalization were analyzed with Mann Whitney U test. Duration of surgery was analyzed with independent student t-test.

*∗*Statistically significant for p<0.05.

**Table 3:** Comparison of postoperative outcomes.

Variable	Plate Fixation Group Mean (SD) N = 36	IM Nail Group Mean (SD) N = 20	P-value
Follow up time, months. (mean ± SD)	38.75 ± 6.83	34.69 ± 7.73	0.06
Union time, months. (mean ± SD)	4.24 ± 0.67	4.47 ± 0.60	0.976
CMS (mean ± SD)	82.40 ± 16.84	77.58 ± 12.96	0.246
DASH score (mean ± SD)	17.46 ± 11.05	20.86 ± 11.63	0.299
Complication rate (mean ± SD)	13.8%	10%	0.097

CMS = Constant-Murley score; DASH = Disabilities of the Arm, Shoulder and Hand; SD = Standard Deviation.

All variables were analyzed with independent student t-test. p < 0.05 is considered significant.

## Data Availability

The data used to support the findings of this study are available from the corresponding author upon request.
